# Collaborative ring trial of two real-time PCR assays for the detection of porcine- and chicken-derived material in meat products

**DOI:** 10.1371/journal.pone.0206609

**Published:** 2018-10-29

**Authors:** Qiang Wang, Yicun Cai, Yuping He, Litao Yang, Liangwen Pan

**Affiliations:** 1 Technical Center for Animal, Plant and Food Inspection and Quarantine, Shanghai Entry-Exit Inspection and Quarantine Bureau of China, Pudong New Area, Shanghai, China; 2 School of Life Science and Biotechnology, Shanghai Jiao Tong University, Minhang Area, Shanghai, China; University of Helsinki, FINLAND

## Abstract

In this study, we describe an inter-laboratory collaborative ring trial validation of species-specific TaqMan real-time PCR assays for the detection of porcine- and chicken-derived materials in meat products. We comprehensively evaluated the performance of these assays in different environments and situations. This validation included the participation of thirteen laboratories across Europe and Asia. The results from the thirteen participating laboratories were analyzed to determine the specificity, accuracy, false positive rate, limit of detection (LOD), and probability of detection (POD) of the developed methods. These results indicated that the methods developed to detect porcine- and chicken-derived materials in meat products are robust and repeatable. The false positive and false negative rates were both 0%, and the LOD was determined to be five copies/reaction. The laboratory standard deviation (σ_L_) was 0.30 for both detection methods, indicating that the developed methods are suitable for detection and identification of the porcine- and chicken-derived materials in meat products.

## Introduction

The identification and quantification of animal materials derived from different species in food and feedstuffs plays an important role in the supervision of food safety. Many infectious zoonotic diseases, including swine influenza and avian influenza, can be easily transmitted to human or animal through consumption of contaminated porcine- or chicken-derived food or feedstuffs [1, 2]. The potential to spread these diseases poses a grave threat to humans and animals health [3], and many countries have banned importing food and feed products that contain porcine or chicken materials from regions affected by these diseases. Additionally, incidences of adulteration of food products with porcine or chicken materials can attract significant public attention and can cause problems in various social and religious contexts [4, 5]. For example, the recent scandals with horse meat and halal beef burgers adulterated with pork shocked entire countries [6–8]. Therefore, many countries and regions, including the European Union, requires that food and feedstuffs must be labelled with accurate and detailed information regarding the composition of all animal material [9]. Biological surveillance of the adulteration of animal materials is a major challenge for governmental agencies [10]. However, there are no widely acknowledged and recognized ISO (International Organization for Standardization) standards for the detection of animal material in foodstuffs. In order to establish national and international standards for such analytical methods, the performance of these methods in different environments and situations need to be comprehensively evaluated. Therefore, in order to foster public health, fair-trade economy, and to prevent antagonism of religious groups, it is necessary and urgent to establish recognized international standards for animal material detection in foodstuffs.

In order to identify the species origin of animal materials in food and feedstuffs, DNA based PCR techniques have become important tools [10, 11]. DNA contains species-specific information and indisputably describes biological diversity, and is an ideal target for molecular detection and identification of species-specific biological products [12, 13]. Many methods have been developed and applied to identify biological materials from specific animal species based on DNA analysis [11, 14–20]. Presently, real-time PCR employing TaqMan probes is a well-established technique, and has been widely used due to its ease of operation, one-step procedure, good specificity, high accuracy, high efficiency, high sensitivity, and a low false-positive rate [10, 13, 21–26].

In this study, the porcine- and chicken-specific TaqMan real-time PCR assays were developed to detect porcine and chicken components in meat products. The performance of these assays was comprehensively evaluated in different environments and situations. We conducted an international collaborative ring trial and determined that these assays exhibit good specificity, high accuracy and sensitivity, and low false positive rates.

## Materials and methods

### 2.1 Meat materials and DNA extraction

Fresh porcine (*Sus*. *scrofa*) and chicken (*Gallus*. *gallus*) meat was purchased from small local slaughterhouses (Benxi, Liaoning, China), and the entire slaughtering process was carefully monitored. Reference DNA from other species, purchased from Zyagen Laboratories (San Diego, CA, USA), was used to verify the specificity of the assays. Since muscle tissue is the most common animal tissue used for cooking or as a component material of other products [27], porcine and chicken muscle tissue was used to develop the analytical methods in this study. Tissue samples were minced, dried in a baking oven (UFE500AO, Memeert, Germany) at 80°C for 72 h, then milled into superfine powder in liquid nitrogen using a Freezer Mixer (6850 freezer/mill, SPEX Sample Prep, USA). Meat powders were used as reference material for DNA extraction and detection procedures. Genomic DNA was extracted using a phenol/chloroform extraction method [28]. Briefly, 100 mg meat powder sample was mixed with 800 μl extraction buffer and 20 μl proteinase K solution (20 mg/ml, Tiangen, China). After incubation 65°C for 60 minutes, with occasional vigorous shaking, an equal volume of phenol-chloroform isoamyl alcohol was added to each sample. Samples were thoroughly mixed and centrifuged at 5000 × g for 15 min. The resulting supernatant was collected, and an equal volume of chloroform/isoamyl alcohol was added, mixed, and centrifuged at 5000 × g for 10 min. The aqueous layer was transferred to a clean tube, and 2.5 volumes of ice-cold 96% ethanol and one-tenth volume of 3 M potassium acetate (pH 5.2) were added. Samples were mixed, incubated at -20°C for 30 min, and centrifuged at 5000 × g for 30 min. The supernatant was discarded and the pellet was washed twice with 800 μl 70% ethanol. Following centrifugation at 5000 × g for 15 min, the pellet was air-dried and resuspended in 100 μl DNAse-and RNAse-free water (Invitrogen, USA). The DNA concentration was measured using a NanoVue spectrophotometer (GE Healthcare, UK).

### 2.2 Primers and probes

For the detection of porcine- and chicken-specific material, the *S*. *scrofa* beta-actin (ACTB) gene (GenBank accession number: DQ452569.1) [29] and *G*. *gallus* transforming growth factor beta 3 (TGF-β3) gene (GenBank accession number: AY685072.1) [30] were selected as species-specific target sequences. Primers and TaqMan probes were designed using Primer Express Software version 3.0 supported by Applied Biosystems (Foster City, CA, USA). Primers and probes are listed in Table 1. The specificity and homology of all primers and probes were evaluated by BLAST searches against the entire GenBank database.

**Table 1:** DNA sequence of oligonucleotides used in this study.

Primers and probes	DNA sequence of oligonucleotides	Final concentration (nmol/L)
Porcine-97bp-F	5’-CGTAGGTGCACAGTAGGTCTGAC-3’	400
Porcine-97bp-R	5’-GGCCAGACTGGGGACATG-3’	400
Porcine-97bp-P	5’-[FAM]-CCAGGTCGGGGAGTC-[NFQ-MGB]-3’	200
Chicken-77bp-F	5’-CAGCTGGCCTGCCGGC-3’	400
Chicken-77bp-R	5’-GCCCAGTGGAATGTGGTATTCA-3’	400
Chicken-77bp-P	5’-[FAM]-TGCCACTCCTCTGCACCCAGTGC-[TAMRA]-3’	200

FAM, 6-Carboxyfluorescein; MGB, Minor Groove Binder (non-fluorescent chromophore); TAMRA, 6-Carboxytetramethylrhodamine (non-fluorescent chromophore).

### 2.3 Plasmid DNA

**Fig 1:**
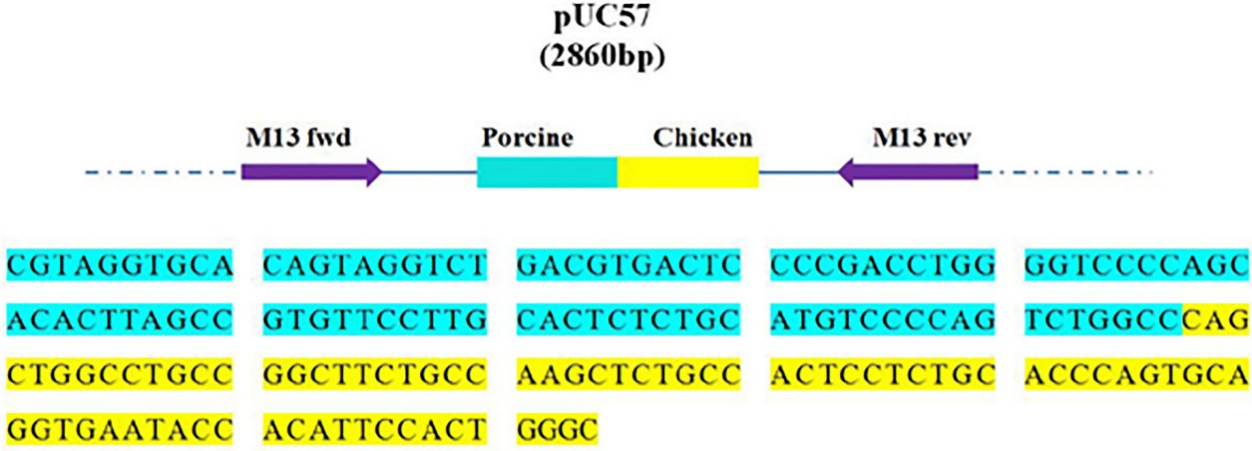
Porcine and chicken target DNA sequence of nucleotides and annotation of the insertion in plasmid pUC57. The plasmid was sequenced to ensure that only one copy of the porcine and chicken target DNA sequence was inserted. Inserted sequences were in line with expectations, and no deletion or insertion mutations were found.

A recombinant pUC57 plasmid (Sangon Biotech, Shanghai, China), including porcine- and chicken-specific DNA fragments was constructed (Fig 1) and was used as a calibrator to determinate the LOD and POD of the real-time PCR assays. Sequencing of the pUC57 plasmid confirmed the insertion of a single copy of each species-specific DNA fragment, and no deletion or insertion mutations were found. The absolute copy number of the plasmid was determined by analyzing the two target DNA fragments using a Bio-Rad QX200 droplet digital PCR system (Hercules, CA, USA).

### 2.4 Real-time PCR

Real-time PCR was performed with in a 25 μl reaction volume, consisting of 1 μl of each primer (10 μmol/L), 0.5 μl probe (10 μmol/L), 12.5 μl real-time PCR Master Mix (Bio-Rad, Hercules, CA, USA), 5 μl nuclease- and protease-free water (Thermo Scientific, Salt Lake City, UT, USA), and 5 μl of sample DNA or plasmid DNA. For amplification, different real-time PCR instruments and software versions (ABI7300 v 1.3.1, ABI7500 v 2.3, ABI ViiA7 v 1.2, ABI7900HT v 2.3, Bio-Rad CFX96 v 3.1 and Roche Light Cycler 480 v 1.5) were used in the collaborative trial. The thermal cycling program included an initial denaturation step at 95°C for 10 min, followed by 45 cycles of 15 s at 95°C for denaturation, and 60 s at 60°C for annealing and extension. The fluorescent signal was collected after the extension step in each cycle.

### 2.5 Evaluation of method specificity

The specificity of the methods, including assay exclusivity and inclusivity, were determined according to established practices [27, 31]. To assess exclusivity, DNA samples from porcine (*S*. *scrofa*), chicken (*G*. *gallus*), and 25 non-target animal species (Table 2) were used. The inclusivity for porcine or chicken DNA was tested using five breeding lines representing each species. The five breeding lines for evaluating inclusivity for DNA from porcine sources were *S*. *scrofa* Bamei, *S*. *scrofa* Meishan, *S*. *scrofa* Qingping, *S*. *scrofa* Tongcheng and *S*. *scrofa* Landrace. The five breeding lines for evaluating inclusivity for DNA from chicken sources were *G*. *gallus* Langshan, *G*. *gallus* Luyuan, *G*. *gallus* Xiaoshan, *G*. *gallus* Yangshan and *G*. *gallus* White Recessive Rocks. The concentration of all reference DNA samples used in the exclusivity and inclusivity tests was 20 ng/μl. In addition, the Sanger DNA sequencing method [32] was utilized to determine the species identity of all reference DNA samples used in this project; this enabled us to rule out heterogeneous contamination and to guarantee the reliable specificity of test results.

**Table 2:** Exclusivity of the porcine and chicken material detection method.

Species tested	Porcine	Chicken
1. Porcine (*Sus scrofa*)	+	-
2. Chicken (*Gallus gallus*)	**-**	+
3. Donkey (*Equus asinus*)	-	-
4. Sheep (*Ovis aries*)	-	**-**
5. Goat (*Capra hircus*)	-	-
6. Cattle (*Bos taurus*)	-	-
7. Horse (*Equus caballus*)	-	-
8. Elk (*Cervus canadensis*)	-	-
9. Buffalo (*Bubalus bubalus*)	-	-
10. Rabbit (*Oryctolagus cunicul*us)	-	-
11. Indian Zebu (*Bos indicus*)	-	-
12. Duck (*Anas platyrhynchos*)	-	-
13. Goose (*Anser anser*)	-	-
14. Turkey (*Meleagris gallopavo*)	-	-
15. Ostrich (*Struthio camelus*)	-	-
16. Pigeon (*Columba livia*)	-	-
17. Quail (*Coturnix coturnix*)	-	-
18. Pheasant (*Phasianus colchicus*)	-	-
19. Monkey (*Macaca mulatta*)	-	-
20. Mouse (*Mus musculus*)	-	-
21. Rat (*Rattus norvegicus*)	-	-
22. Goldfish (*Carassius auratus*)	-	-
23. Carp (*Cyprinus carpio*)	-	-
24. Trout (*Onchorhynchus mykiss*)	-	-
25. Camel (*Camelus bactrianus*)	-	-
26. Cat (*Felis catus*)	-	-
27. Dog (*Canis familiaris*)	-	-

+, represent positive results; -, represent negative results.

### 2.6 Collaborative trial

The collaborative trial was organized by the Technical Center for Animal, Plant and Food Inspection and Quarantine, Shanghai Entry-Exit Inspection and Quarantine Bureau of China (SHCIQ), and was implemented from Nov. 2016 to Jan. 2017. A total of thirteen laboratories from five countries (France, Germany, Malaysia, Portugal, and China) were invited and participated in the collaborative trial. Each laboratory received a package including the following samples and reaction reagents needed for the validation test: 1) twelve DNA samples with a volume of 50 μl (C1-C12, sample codes randomly assigned) were provided for porcine-specific detection, including six tubes of porcine genomic DNA samples (2 copies/μl) and six tubes of horse genomic DNA samples (4 copies/μl); 2) twelve DNA samples with a volume of 50 μl (D1-D12, sample codes randomly assigned) were provided for chicken-specific detection, including six tubes of chicken genomic DNA samples (2 copies/μl) and six tubes of horse genomic DNA samples (4 copies/μl); 3) one pUC57 plasmid DNA sample with a volume of 50 μl (1000 copies/μl) was provided for LOD and POD test; 4) one salmon sperm DNA sample with a volume of 50 μl (10 mg/ml; Invitrogen, Carlsbad, USA, CA 92008) was provided; participants were requested to dilute the salmon sperm DNA samples to 20 ng/μl with ddH_2_O for further experiments; 5) two bottles of 2 × 5 ml TaqMan Gene Expression Master Mix (Foster City, USA, CA 94404) were provided; and 6) two pairs of primers and probes, purchased from Sangon Biotech (Shanghai, China) were provided in dry powder form, and were diluted to 10 μmol/L with ddH_2_O before using.

All packages were transported on ice by air to each participating laboratory. All participating laboratories received an operation protocol and a results report sheet. The participants were requested to operate strictly according to the operation protocol (S1 File), and to report any deviation from the protocol which may have occurred during the experimental operation.

For the false-positive and false-negative rates test, twelve blind samples (six positive and six negative samples) were evaluated by each detection method. The effective concentrations of positive and negative samples were 10 and 20 copies/reaction, respectively. The total number of reactions for each method was 156 among the 13 participating labs.

To evaluate limit of detection (LOD) and probability of detection (POD), Microsoft Excel 2010 and a new mathematical statistical model [33] were used, respectively. The pUC57 plasmid DNA was serially diluted from 1000 copies/μl to 4, 2, 1, 0.4, 0.2, 0.1 and 0.02 copies/μl using the salmon sperm DNA solution (20 ng/μl). Six replicates were performed for each dilution. The total number of reactions was 43 (including a blank control) for each method in each participating lab.

## Results and discussion

Each of the thirteen participating laboratories returned all experimental data (Ct values) in a timely manner (S1 File). Although the participants were widely distributed in different countries, with different experimental operators, laboratory environments, and instrumentation, consistent experimental results were reported from all labs. None of the participating laboratories reported any experimental problems or obvious deviations during the sample preparation and detection procedures, confirming that the assays are widely applicable and the methodology is straightforward.

### 3.1 Specificity

A comprehensive combination of theoretical (bioinformatics and sequence alignment) and practical tests was used to evaluate the specificity of the methods. The theoretical specificity of porcine- and chicken-specific primers and probes was analyzed by BLASTN similarity searches against the entire NCBI genome database. Sequence alignment of the candidate primers and probes with homologous sequences from other common animal species demonstrated no likelihood for significant cross reaction with other species. In practice, the exclusivity was tested using a broad range of DNA samples from 27 different animal species, including two target species and 25 non-target species. Only the expected positive signals were detected from porcine and chicken target DNA amplification, and no visible amplification signals were detected for other species (Table 2). The inclusivity of porcine and chicken was tested on samples from five breeding lines for each species, and the expected target amplification signals were detected in breeds of the corresponding species (Table 3). These results confirmed the high specificity of the porcine- and chicken-specific detection methods, both in theory and in practice.

**Table 3:** Inclusivity of the porcine and chicken material detection method.

Breeds tested	Porcine	Chicken
*S*. *scrofa* Bamei	+	
*S*. *scrofa* Meishan	+	
*S*. *scrofa* Qingping	+	
*S*. *scrofa* Tongcheng	+	
*S*. *scrofa* Landrace	+	
*G*. *gallus* Langshan		+
*G*. *gallus* Luyuan		+
*G*. *gallus* Xiaoshan		+
*G*. *gallus* Yangshan		+
*G*. *gallus* White Recessive Rocks		+

+, represent positive results.

### 3.2 Robustness

In this collaborative study, six different real-time PCR cycler brands and/or models were used by the different participating laboratories, and the participants reported no abnormal results. Furthermore, in subsequent data analysis, no significant differences were identified, indicating that the detection method is robust and reliable.

### 3.3 False-positive and false-negative rates

For each detection method, the false-positive and false-negative rates were calculated based on the results of the 156 PCR reactions from the thirteen participating laboratories. As anticipated, all positive samples were correctly identified as positive, and all negative samples were confirmed as negative, no abnormal results were reported. The false positive and false negative rates were 0% (Table 4) for both detection methods, indicating the high reliability the real-time PCR detection methods developed here.

**Table 4:** Summary of false-positive and false-negative rates.

Sample	Porcine	Chicken
Number of laboratories	13	13
Number of laboratories evaluated	13	13
Number of samples per laboratory	12	12
Total number of samples	156	156
Number of accepted results	156	156
Number of samples containing the target sequence	78	78
Target sequence concentration (copies/μl PCR)	2	2
Number of samples not containing the target sequence	78	78
Number of positive results for positive samples	78	78
Number of negative results for negative samples	78	78
Number of false-negative results	0	0
False-positive rate (%)	0	0
False-negative rate (%)	0	0

### 3.4 LOD and POD

In this study, LOD was defined as the lowest concentration at which 95% of replicates reported positive qualitative results [34]. For each detection method, a total of 78 results were obtained for each dilution level from the thirteen participating laboratories. Based on the collaborative ring trial results, the LOD_95%_ for the porcine or chicken detection methods was determined with a value of 5 copies/reaction (Table 5).

**Table 5:** Summary of collaborative trial results for the LOD_95%_ test.

Concentration	Porcine		Chicken	
Copies/tube	P/T	P (%)	P/T	P (%)
20	78/78	100	78/78	100
10	78/78	100	78/78	100
5	78/78	100	78/78	100
2	72/78	92.3	69/78	88.5
1	49/78	62.8	42/78	53.8
0.5	30/78	38.5	25/78	32.1
0.1	7/78	9.0	6/78	7.7

P, number of positive results; T, total number of tests; P (%), positive rate.

For the POD of porcine and chicken detection methods across laboratories, qualitative data from all PCR experiments at different dilutions were used to estimate the laboratory standard deviation σ_L_, and the theoretical median copy number_._ The σ_L_ represents the relative between-laboratory variability at POD = 0.95. A value of 0.3 was obtained for the laboratory standard deviation of both detection methods, and the LOD_95%_ (in copies) of the theoretical median laboratory was 3.1 and 3.3, respectively (Table 6). The LOD_95%_ and POD test results indicate the high sensitivity for porcine and chicken detection methods.

**Table 6:** Summary of collaborative trial results for the POD test.

Parameters	Porcine	Chicken
Number of laboratories	13	13
Number of PCR replicates per dilution level	6	6
Mean probability of detection across laboratories (*LPOD*)	0.78	0.74
95% confidence interval for *LPOD*	0.67–0.87	0.63–0.84
Slope b relative to the ideal *POD* curve (b = 1)	1.17	1.18
Laboratory standard deviation σ_L_	0.30	0.30
*LOD*_*95%*_ (in copies) of the theoretical median laboratory	3.1	3.3

## Conclusion

Accurate detection and identification of animal materials from different species in food and feedstuffs plays an important role in food safety supervision. The development of standardized methods for detection and identification of porcine and chicken material will greatly facilitate the regulation of meat products and reduce instances of meat adulteration. In this study we describe the development and validation of species-specific real-time PCR methods for porcine and chicken material in meat products, which will assist in identifying fraudulent and/or mislabeled products of animal origin in meat products. The validation of these methods by a collaborative ring trial demonstrates their broad applicability and robustness of use. The high specificity and low false positive/negative rates demonstrate the reliability and applicability of these real-time PCR detection methods, and the LOD_95%_ and POD analyses demonstrate the high sensitivity of these methods. In conclusion, these methods demonstrate excellent and reliable performance for the detection and identification of porcine- and chicken-derived materials in meat products.

## Supporting information

S1 File Protocol and results. Protocol and results of inter-laboratory collaborative validation trial for species specific real-time PCR assays of porcine and chicken-derived material in meat products.
(DOCX)
