# Supplementary material for: Collaborative ring trial of two real-time PCR assays for the detection of porcine- and chicken-derived material in meat products
Source: PLoS One. 2018 Oct 29;13(10):e0206609. doi: 10.1371/journal.pone.0206609 (PMC6205609; doi:10.1371/journal.pone.0206609)
Supplement: S1 File — Protocol and results of inter-laboratory collaborative validation trial for species specific real-time PCR assays of porcine and chicken-derived material in meat products. (DOCX) [file pone.0206609.s001.docx]

**Protocol and results of inter-laboratory collaborative validation trial for species specific real-time PCR assays of porcine and chicken-derived material in meat products**

1. **Description**

This file is designed to conduct an inter-laboratory validation trial for the detection of porcine and chicken-derived material in meat products by real-time PCR targeting on the taxon specific genomic DNA fragment. In order to further verify the reliability and applicability of these methods, the Technical Center for Animal, Plant and Food Inspection and Quarantine of Shanghai Entry-Exit Inspection and Quarantine Bureau managed to launch the inter-laboratory ring trial.

1. **Materials and document provided**

**Note: After receiving the validated materials, the primers, probes, samples DNA, plasmids DNA and Sonicated salmon sperm DNA must be stocked in –20 °C, and the TaqMan Master mixes should be stocked in 4 °C.**

- 1. **Paper documents**
     1. Instructions of inter-laboratory trial.
     2. Protocol of inter-laboratory trial.
     3. Receipt form.
     4. Results report sheet.
  2. **Primers and probes**

**Table 1 Primers and probes**

| **Species** | **Name** | **DNA sequence of the oligo nucleotide** | **OD** | **Adding ddH_2_O volume (μl)** | **Concentration**  **(µM)** |
| --- | --- | --- | --- | --- | --- |
| Porcine  (*Sus scrofa*) | Porcine-97bp-F | 5’-CGTAGGTGCACAGTAGGTCTGAC-3’ | 1 | 440 | 10 |
|  | Porcine-97bp-R | 5’-GGCCAGACTGGGGACATG-3’ | 1 | 560 | 10 |
|  | Porcine-97bp-P | 5’-[FAM]-CCAGGTCGGGGAGTC-[MGB]-3’ | 1 | 690 | 10 |
| Chicken  (*Gallus gallus*) | Chicken-77bp-F | 5’- CAGCTGGCCTGCCGGC-3’ | 1 | 730 | 10 |
|  | Chicken-77bp-R | 5’- GCCCAGTGGAATGTGGTATTCA-3’ | 1 | 460 | 10 |
|  | Chicken-77bp-P | 5’-[FAM]-TGCCACTCCTCTGCACCCAGTGC-[TAMRA]-3’ | 1 | 510 | 10 |

- 1. **Samples**
     1. **Sample DNA for real-time PCR false positive and false negative tests**

The real-time PCR false positive and false negative tests should be carried out with 6 positive and 6 negative blind samples. Each participant will receive 24 tubes of sample DNA coded C1~C12 and D1~D12. The information of the sample DNA are shown in Table 2.

**Table 2­­­­­­­ The information of the sample DNA**

| **Samples DNA** | **Concentration**  **(copies/μl)** | **Extracted from** | **Positive samples number** | **Negative samples number** | **Tested for** |
| --- | --- | --- | --- | --- | --- |
| C1~C12 | 10 or 20 | Porcine or horse meat | 6 | 6 | Porcine PCR |
| D1~D12 | 10 or 20 | Chicken or horse meat | 6 | 6 | Chicken PCR |

- - 1. **Plasmid DNA for real-time PCR *LOD* and *POD* tests**

The concentration of the provided plasmid DNA is 1000 copies/μl. The plasmid containing the porcine and chicken specific detection fragments is used for *LOD* and *POD* tests of the real-time PCR method.

The provided plasmid DNA should be diluted using sonicated salmon sperm DNA (20 ng/μl) from 1000 copies/μl to seven concentrations of 4, 2, 1, 0.4, 0.2, 0.1 and 0.02 copies/μl. For every method test, 6 replicates for each concentration point should be set up.

- 1. **Regents**
     1. 2×Taqman Gene Expression Master Mix 5 ml (LifeScienceseries equipment like 7300, 7500 or ViiA^TM^7).
     2. Sonicated salmon sperm DNA (10 mg/ml) 50 μl.

1. **Required reagents and Instruments but not provided**
   1. Double distilled, sterile H_2_O (ddH_2_O), or equivalent.
   2. Real-time PCR device.
   3. Vortex.
   4. –20 ºC freezer and 4 °C refrigerator.
   5. Vessel racks suitable of maintaining 4 °C.
   6. 1.5-ml Eppendorf tubes.
   7. 200-μl PCR reaction tubes or 96-well plates.
   8. Variable speed micro centrifuge capable of 15,000×g.
   9. Combination of micropipettes and aerosol-barrier pipette tips capable of transferring volumes from 1 to 1000µl.
   10. Disposable rubber gloves.
2. **Protocols**
   1. **Preparation of the working primer and probe solutions**

The primers and probes were provided in dry powder. Centrifuge the primer and probe tubes at a speed of 7,000×g for 3 minutes, add ddH_2_O to the dry primers and probes according to the Table 1. Vortex the solutions for 30 seconds vigorously, then place it in 4°C refrigerator for 1 hour. Centrifuge it at a speed of 3,000×g after vortex again. The 10 µM working primer and probe solutions are prepared.

- 1. **Protocol for false positive and false negative rates test**

**4.2.1 PCR setup**

For porcine real-time PCR detection method, the sample DNA coded C1~C12 and ddH_2_O (blank control) as the PCR DNA templates, Porcine-97bp-F, Porcine-97bp-R and Porcine-97bp-P as the primers and probe set are used.

For chicken real-time PCR detection method, the sample DNA coded D1~D12 and ddH_2_O (blank control) as the PCR DNA templates, Chicken-77bp-F, Chicken-77bp-R and Chicken-77bp-P as the primers and probe set are used.

Since the total reaction tubes are 13 (1 for blank control) for each of the method test. We recommend to make each of the pre-mixture tube with 15 reaction volumes (2 more reaction volume to ensure the sufficient pre-mixture).

Place two 1.5-ml eppendorf tubes on ice, add the reagents to each of the tube according to the order shown in the Table 3 respectively, except for the DNA. After finishing the adding, vortex the master tube gently and centrifuge it at the speed of 3,000×g for 30 seconds, then distribute the master mixture to thirteen 200-μl PCR tubes or 96-well plate. Add 5 μl sample DNA solutions to the tubes or plates as shown in the Table 4. Mix the PCR tubes gently, centrifuge temporarily in the micro centrifuge.

**Table 3 Addition of reagents**

| **Reagent** | **Volume per sample (μl)** | **Pre-mixture for**  **15 reactions (2 tubes more) (μl)** |
| --- | --- | --- |
| TaqMan Master mix | 12.5 | 187.5 |
| Forward primer (10 µM) | 1 | 15 |
| Reverse primer (10 µM) | 1 | 15 |
| Probe (10 µM) | 0.5 | 7.5 |
| DNA sample | 5 | --- |
| ddH_2_O | 5 | 75 |
| Final reaction Volume | 25 | --- |

**Table 4 Sample setting order**

|  | **Primers and probes** | | | | | | | | | | | |
| --- | --- | --- | --- | --- | --- | --- | --- | --- | --- | --- | --- | --- |
|  | **Porcine PCR** | | **Chicken PCR** | |  | |  | |  |  |  |  |
|  | **1** | **2** | **3** | **4** | **5** | **6** | **7** | **8** | **9** | **10** | **11** | **12** |
| **1** | C1 | C9 | D1 | D9 |  |  |  |  |  |  |  |  |
| **2** | C2 | C10 | D2 | D10 |  |  |  |  |  |  |  |  |
| **3** | C3 | C11 | D3 | D11 |  |  |  |  |  |  |  |  |
| **4** | C4 | C12 | D4 | D12 |  |  |  |  |  |  |  |  |
| **5** | C5 | ddH_2_O | D5 | ddH_2_O |  |  |  |  |  |  |  |  |
| **6** | C6 | --- | D6 | --- |  |  |  |  |  |  |  |  |
| **7** | C7 | --- | D7 | --- |  |  |  |  |  |  |  |  |
| **8** | C8 | --- | D8 | --- |  |  |  |  |  |  |  |  |

**4.2.2 Temperature-time program**

Place the tubes or plates on the real-time PCR device. PCR reactions are carried out by using the thermal cycling as in the Table 5.

**Table 5 Temperature–timeprogram**

| **Step** | **Parameter** | | **Temperature** | **Time** | **Fluorescence measurement** | **Cycles** |
| --- | --- | --- | --- | --- | --- | --- |
| 1 | Initial Denaturation | | 95°C | 10 min | no | 1 |
| 2 | Amplification | Denaturation | 95°C | 15 sec | no | 45 |
|  |  | Annealing and extension | 60°C | 60 sec | yes |  |

**4.2.3 Results record and report**

The detected results (Ct value) and Detected/Not Detected should be recorded in the Result Report Sheet 1.

**4.****3 Protocol for real-time PCR method *LOD* and *POD* tests**

**4.3.1 Dilution of the salmon sperm DNA**

The salmon sperm DNA (10 mg/ml) should be diluted using ddH_2_O by the participants from 10 mg/ml to 20 ng/μl. The dilution procedure is shown in the Table 6.

**Table 6 Dilution method of salmon sperm DNA**

| **Adding salmon sperm DNA volume (μl)** | **Adding ddH_2_O volume (μl)** | **Diluted concentration (ng/μl)** | **Final volume (μl)** |
| --- | --- | --- | --- |
| 10 | 4990 | 20 | 5000 |

**4.3.2 Dilution of the plasmid DNA for PCR method *LOD* and *POD* tests**

The 1000 copies/μl of plasmid DNA is used for the *LOD* and *POD* tests. A serial dilution method should be prepared by the participants from 1000 copies/μl to 4, 2, 1, 0.4, 0.2, 0.1 and 0.02 copies/μl using the salmon sperm DNA (20 ng/μl). The dilution procedure is shown in the Table 7. We strongly recommend do not use the high speed of vortex.

**Table 7 Dilution method of plasmid DNA for PCR method *LOD* and *POD* tests**

| **Master solution**  **concentration (copies/μl)** | **Adding master volume (μl)** | **Adding salmon sperm DNA (20 ng/μl) volume (μl)** | **Diluted concentration (copies/μl)** | **Final volume (μl)** |
| --- | --- | --- | --- | --- |
| 1000 | 10 | 90 | 100 | 100-24=76 |
| 100 | 24 | 576 | **4** | 600-330=**270** |
| 4 | 300 | 300 | **2** | 600-330=**270** |
| 2 | 300 | 300 | **1** | 600-30=**570** |
| 4 | 30 | 270 | **0.4** | **300** |
| 2 | 30 | 270 | **0.2** | 300-30=**270** |
| 1 | 30 | 270 | **0.1** | **300** |
| 0.2 | 30 | 270 | **0.02** | **300** |

**4.3.3 PCR setup**

The plasmid standard DNA concentrations for *LOD* and *POD* tests are 4, 2, 1, 0.4, 0.2, 0.1 and 0.02 copies/μl. For each concentration level, 6 replicates were tested for every animal species detection method, the total reaction tubes or plates are 43 (1 for blank control with the diluted salmon sperm DNA) for each of the method test. We recommend to make each of the pre-mixture tube with 50 reaction volumes (7 more reaction volumes to ensure the sufficient pre-mixture), and test the porcine and chicken PCR methods in one 96-well plate at the same time.

Place two 1.5-ml Eppendorf tubes on ice, add the reagents to each of the tube according to the order shown in the Table 8 respectively, except for the DNA. After finishing the adding, vortex the master tube gently and centrifuge it at the speed of 3,000×g for 30 seconds, then distribute the master mixture of each tube to forty-three 200-μl PCR reaction tubes or 96-well plate. Add 5µl the plasmid standard DNA solutions to the tubes or plates as shown in the Table 9. Mix the PCR tubes gently, centrifuge temporarily in the micro centrifuge.

**Table 8 Addition of reagents**

| **Reagent** | **Volume per sample (μl)** | **Pre-mixture for**  **50 reactions (7 tubes more) (μl)** |
| --- | --- | --- |
| TaqMan Master mix | 12.5 | 625 |
| Forward primer (10 µM) | 1 | 50 |
| Reverse primer (10 µM) | 1 | 50 |
| Probe (10 µM) | 0.5 | 25 |
| DNA sample | 5 | -- |
| ddH_2_O | 5 | 250 |
| Final reaction Volume | 25 | -- |

**Table 9 Sample setting order (copies/μl)**

|  | **Primers and probes** | | | | | | | | | | | |
| --- | --- | --- | --- | --- | --- | --- | --- | --- | --- | --- | --- | --- |
|  | **Porcine PCR** | | | | | | **Chicken PCR** | | | | | |
|  | **1** | **2** | **3** | **4** | **5** | **6** | **7** | **8** | **9** | **10** | **11** | **12** |
| **1** | 4 | 4 | 4 | 4 | 4 | 4 | 4 | 4 | 4 | 4 | 4 | 4 |
| **2** | 2 | 2 | 2 | 2 | 2 | 2 | 2 | 2 | 2 | 2 | 2 | 2 |
| **3** | 1 | 1 | 1 | 1 | 1 | 1 | 1 | 1 | 1 | 1 | 1 | 1 |
| **4** | 0.4 | 0.4 | 0.4 | 0.4 | 0.4 | 0.4 | 0.4 | 0.4 | 0.4 | 0.4 | 0.4 | 0.4 |
| **5** | 0.2 | 0.2 | 0.2 | 0.2 | 0.2 | 0.2 | 0.2 | 0.2 | 0.2 | 0.2 | 0.2 | 0.2 |
| **6** | 0.1 | 0.1 | 0.1 | 0.1 | 0.1 | 0.1 | 0.1 | 0.1 | 0.1 | 0.1 | 0.1 | 0.1 |
| **7** | 0.02 | 0.02 | 0.02 | 0.02 | 0.02 | 0.02 | 0.02 | 0.02 | 0.02 | 0.02 | 0.02 | 0.02 |
| **8** | --- | --- | --- | --- | --- | blank control | --- | --- | --- | --- | --- | blank control |

**4.3.4 Temperature-time program**

Place the tubes or plates on the real-time PCR device. PCR reactions are carried out by using the thermal cycling as in the Table 5.

1. **Summary of results**

Table 10 shows the summary of the results of all the unknown samples provided by the thirteen laboratories. The false-positive rate or false-negative rate is calculated by 156 PCR results obtained for each series of 12 samples.

For the porcine and chicken PCR methods, all positive samples were correctly tested positive, all negative samples were correctly tested negative resulting in false-positive and false-negative rates of 0% (Table 11).

Table 12 and 13 show the summary of the results of the *LOD_95%_* and *POD* test of porcine and chicken real-time PCR, provided by the thirteen laboratories. The average positive rate of each concentration point was calculated on basis of 78 PCR repetitions.

For the porcine PCR method *LOD_95%_* test, the positive rate of 20, 10, 5, 2, 1, 0,5 and 0,1 copies/reaction concentration points are 100%, 100%, 100%, 92,3%,61,5%, 38,5% and 9,0% respectively (Table 14). The *LOD_95%_* of the porcine PCR methods is at least 5 copies/reaction.

For the chicken PCR method *LOD_95%_* test, the positive rate of 20, 10, 5, 2, 1, 0,5 and 0,1 copies/reaction concentration points are 100%, 100%, 100%, 88,5%, 53,8%, 32,1% and 7,7% respectively (Table 14). The *LOD_95%_* of the chicken PCR method is at least 5 copies/reaction.

On the basis of the qualitative data, statistical modelling for the *POD* across all laboratories was done for the different real-time PCR methods. The laboratory standard deviations σ_L_ are estimated. The σ_L_ values of the porcine and chicken PCR method are both 0,30. The *LOD_95%_* of the theoretical median laboratory of the porcine and chicken PCR method are 3,1 and 3,3 copies respectively (Table 15).

**Table 10 The summary of the results of false-positive and false-negative rates test**

|  | | **Porcine PCR** | | | | | | | | | | | |
| --- | --- | --- | --- | --- | --- | --- | --- | --- | --- | --- | --- | --- | --- |
| **Primers and probe** | | **Porcine-97bp-F/Porcine-97bp-R/Porcine-97bp-P** | | | | | | | | | | | |
| **Sample No.** | | C1 | C2 | C3 | C4 | C5 | C6 | C7 | C8 | C9 | C10 | C11 | C12 |
| **Lab No.** | 1 | - | - | + | - | - | + | + | - | - | + | + | + |
|  | 2 | - | - | + | - | - | + | + | - | - | + | + | + |
|  | 3 | - | - | + | - | - | + | + | - | - | + | + | + |
|  | 4 | - | - | + | - | - | + | + | - | - | + | + | + |
|  | 5 | - | - | + | - | - | + | + | - | - | + | + | + |
|  | 6 | - | - | + | - | - | + | + | - | - | + | + | + |
|  | 7 | - | - | + | - | - | + | + | - | - | + | + | + |
|  | 8 | - | - | + | - | - | + | + | - | - | + | + | + |
|  | 9 | - | - | + | - | - | + | + | - | - | + | + | + |
|  | 10 | - | - | + | - | - | + | + | - | - | + | + | + |
|  | 11 | - | - | + | - | - | + | + | - | - | + | + | + |
|  | 12 | - | - | + | - | - | + | + | - | - | + | + | + |
|  | 13 | - | - | + | - | - | + | + | - | - | + | + | + |
|  | | **Chicken PCR** | | | | | | | | | | | |
| **Primers and probe** | | **Chicken-77bp-F/Chicken-77bp-R/Chicken-77bp-P** | | | | | | | | | | | |
| **Sample No.** | | D1 | D2 | D3 | D4 | D5 | D6 | D7 | D8 | D9 | D10 | D11 | D12 |
| **Lab No.** | 1 | + | - | - | + | + | - | - | + | + | + | - | - |
|  | 2 | + | - | - | + | + | - | - | + | + | + | - | - |
|  | 3 | + | - | - | + | + | - | - | + | + | + | - | - |
|  | 4 | + | - | - | + | + | - | - | + | + | + | - | - |
|  | 5 | + | - | - | + | + | - | - | + | + | + | - | - |
|  | 6 | + | - | - | + | + | - | - | + | + | + | - | - |
|  | 7 | + | - | - | + | + | - | - | + | + | + | - | - |
|  | 8 | + | - | - | + | + | - | - | + | + | + | - | - |
|  | 9 | + | - | - | + | + | - | - | + | + | + | - | - |
|  | 10 | + | - | - | + | + | - | - | + | + | + | - | - |
|  | 11 | + | - | - | + | + | - | - | + | + | + | - | - |
|  | 12 | + | - | - | + | + | - | - | + | + | + | - | - |
|  | 13 | + | - | - | + | + | - | - | + | + | + | - | - |

Note: +: positive result; -: negative result.

**Table 11 Analysis of results of the collaborative trial for false positive and false negative rates test**

| Sample | Porcine | Chicken |
| --- | --- | --- |
| Primer and probe | Porcine-97bp-F  Porcine-97bp-R  Porcine-97bp-P | Chicken-77bp-F  Chicken-77bp-R  Chicken-77bp-P |
| Year | 2016-2017 | 2016-2017 |
| Number of laboratories | 13 | 13 |
| Number of laboratories that have been evaluated | 13 | 13 |
| Number of samples per laboratory | 12 | 12 |
| Number of total samples | 156 | 156 |
| Number of accepted results | 156 | 156 |
| Number of samples containing target sequence | 78 | 78 |
| Target sequence concentration in PCR test (copies**/**μl PCR) | 2 | 2 |
| Number of samples not containing the target sequence | 78 | 78 |
| Number of positive results for positive samples | 78 | 78 |
| Number of negative results for negative samples | 78 | 78 |
| Number of false-negative | 0 | 0 |
| False-positive rate (%) | 0 | 0 |
| False-negative rate (%) | 0 | 0 |

**Table 12 The summary of the results of *LOD_95%_* and *POD* tests of Porcine PCR method**

|  | | **Lab No.** | | | | | | | | | | | | |
| --- | --- | --- | --- | --- | --- | --- | --- | --- | --- | --- | --- | --- | --- | --- |
|  | **Target copies/reaction** | **1** | **2** | **3** | **4** | **5** | **6** | **7** | **8** | **9** | **10** | **11** | **12** | **13** |
| **Porcine PCR** | **20** | + | + | + | + | + | + | + | + | + | + | + | + | + |
|  | **20** | + | + | + | + | + | + | + | + | + | + | + | + | + |
|  | **20** | + | + | + | + | + | + | + | + | + | + | + | + | + |
|  | **20** | + | + | + | + | + | + | + | + | + | + | + | + | + |
|  | **20** | + | + | + | + | + | + | + | + | + | + | + | + | + |
|  | **20** | + | + | + | + | + | + | + | + | + | + | + | + | + |
|  | **10** | + | + | + | + | + | + | + | + | + | + | + | + | + |
|  | **10** | + | + | + | + | + | + | + | + | + | + | + | + | + |
|  | **10** | + | + | + | + | + | + | + | + | + | + | + | + | + |
|  | **10** | + | + | + | + | + | + | + | + | + | + | + | + | + |
|  | **10** | + | + | + | + | + | + | + | + | + | + | + | + | + |
|  | **10** | + | + | + | + | + | + | + | + | + | + | + | + | + |
|  | **5** | + | + | + | + | + | + | + | + | + | + | + | + | + |
|  | **5** | + | + | + | + | + | + | + | + | + | + | + | + | + |
|  | **5** | + | + | + | + | + | + | + | + | + | + | + | + | + |
|  | **5** | + | + | + | + | + | + | + | + | + | + | + | + | + |
|  | **5** | + | + | + | + | + | + | + | + | + | + | + | + | + |
|  | **5** | + | + | + | + | + | + | + | + | + | + | + | + | + |
|  | **2** | + | + | + | + | + | - | + | + | + | + | + | + | + |
|  | **2** | - | + | + | + | + | + | + | + | + | + | + | + | + |
|  | **2** | - | + | + | + | + | + | + | + | + | + | + | + | + |
|  | **2** | + | + | - | + | + | + | + | + | - | + | + | + | + |
|  | **2** | + | + | + | + | + | + | + | + | + | + | + | + | + |
|  | **2** | + | + | + | + | + | + | + | + | + | + | - | + | + |
|  | **1** | - | - | + | + | + | - | - | + | + | - | - | + | + |
|  | **1** | - | - | - | - | + | + | - | + | - | + | + | - | + |
|  | **1** | - | + | - | + | + | + | + | + | + | - | + | + | + |
|  | **1** | - | + | - | + | + | + | - | - | + | - | - | - | + |
|  | **1** | - | + | + | + | + | + | - | + | - | + | - | + | + |
|  | **1** | + | + | + | + | + | + | - | - | + | + | - | + | + |
|  | **0.5** | - | + | - | + | + | + | - | - | - | + | - | + | - |
|  | **0.5** | - | + | - | - | + | - | - | - | + | - | + | - | - |
|  | **0.5** | - | + | - | + | - | + | - | - | - | + | - | - | + |
|  | **0.5** | - | + | + | + | + | + | - | - | + | + | - | - | - |
|  | **0.5** | - | - | + | + | + | - | - | - | - | - | - | - | + |
|  | **0.5** | - | - | + | - | + | - | - | - | + | + | - | - | - |
|  | **0.1** | - | - | - | - | - | - | - | - | - | - | - | + | - |
|  | **0.1** | - | - | - | - | - | - | - | - | - | - | - | - | + |
|  | **0.1** | - | - | - | - | + | - | - | - | - | + | - | - | - |
|  | **0.1** | - | - | - | - | + | - | - | - | - | - | + | - | - |
|  | **0.1** | - | - | - | - | - | - | - | - | - | - | - | - | - |
|  | **0.1** | - | - | - | + | - | - | - | - | - | - | - | - | - |

Note: +: positive result; -: negative result.

**Table 13 The summary of the results of *LOD_95%_* and *POD* tests of Chicken PCR method**

|  | | **Lab No.** | | | | | | | | | | | | |
| --- | --- | --- | --- | --- | --- | --- | --- | --- | --- | --- | --- | --- | --- | --- |
|  | **Target copies/reaction** | **1** | **2** | **3** | **4** | **5** | **6** | **7** | **8** | **9** | **10** | **11** | **12** | **13** |
| **Chicken PCR** | **20** | + | + | + | + | + | + | + | + | + | + | + | + | + |
|  | **20** | + | + | + | + | + | + | + | + | + | + | + | + | + |
|  | **20** | + | + | + | + | + | + | + | + | + | + | + | + | + |
|  | **20** | + | + | + | + | + | + | + | + | + | + | + | + | + |
|  | **20** | + | + | + | + | + | + | + | + | + | + | + | + | + |
|  | **20** | + | + | + | + | + | + | + | + | + | + | + | + | + |
|  | **10** | + | + | + | + | + | + | + | + | + | + | + | + | + |
|  | **10** | + | + | + | + | + | + | + | + | + | + | + | + | + |
|  | **10** | + | + | + | + | + | + | + | + | + | + | + | + | + |
|  | **10** | + | + | + | + | + | + | + | + | + | + | + | + | + |
|  | **10** | + | + | + | + | + | + | + | + | + | + | + | + | + |
|  | **10** | + | + | + | + | + | + | + | + | + | + | + | + | + |
|  | **5** | + | + | + | + | + | + | + | + | + | + | + | + | + |
|  | **5** | + | + | + | + | + | + | + | + | + | + | + | + | + |
|  | **5** | + | + | + | + | + | + | + | + | + | + | + | + | + |
|  | **5** | + | + | + | + | + | + | + | + | + | + | + | + | + |
|  | **5** | + | + | + | + | + | + | + | + | + | + | + | + | + |
|  | **5** | + | + | + | + | + | + | + | + | + | + | + | + | + |
|  | **2** | - | + | + | + | + | + | + | - | + | + | + | + | + |
|  | **2** | - | + | + | + | + | + | + | - | - | + | + | + | + |
|  | **2** | + | + | - | + | + | + | + | + | + | + | + | + | + |
|  | **2** | + | + | + | + | + | + | + | - | + | + | + | + | + |
|  | **2** | - | + | + | + | + | + | + | + | + | + | + | + | + |
|  | **2** | + | - | + | + | + | + | + | + | + | + | + | + | + |
|  | **1** | - | + | - | + | - | + | - | - | - | - | + | + | + |
|  | **1** | - | - | + | - | + | + | - | - | + | + | + | + | + |
|  | **1** | - | + | - | + | - | - | + | - | + | - | + | + | + |
|  | **1** | - | + | - | + | + | + | - | - | - | + | + | + | + |
|  | **1** | - | - | + | + | + | + | + | - | + | + | - | + | + |
|  | **1** | - | - | - | + | + | - | - | - | + | - | - | + | - |
|  | **0.5** | - | - | - | - | + | + | - | - | + | + | + | - | - |
|  | **0.5** | - | + | - | + | - | - | - | - | - | - | + | + | + |
|  | **0.5** | - | + | - | + | - | + | - | - | - | - | - | + | + |
|  | **0.5** | - | - | - | - | - | - | - | - | + | + | - | - | - |
|  | **0.5** | - | - | - | - | - | + | - | - | + | - | - | - | - |
|  | **0.5** | - | - | + | + | + | - | - | - | + | - | - | + | + |
|  | **0.1** | - | - | - | - | - | - | - | - | - | - | - | - | + |
|  | **0.1** | - | - | - | - | - | - | - | - | - | + | - | - | - |
|  | **0.1** | - | - | - | - | - | - | - | - | - | - | - | - | - |
|  | **0.1** | - | - | - | - | - | - | - | - | - | + | - | - | - |
|  | **0.1** | - | - | - | - | - | - | - | - | + | - | - | + | - |
|  | **0.1** | - | - | - | + | - | - | - | - | - | - | - | - | - |

Note: +: positive result; -: negative result.

**Table 14 Analysis of results of the collaborative trial for *LOD_95%_* test**

|  | **Porcine** | | **Chicken** | |
| --- | --- | --- | --- | --- |
| **Target copies/PCR** | **P/T** | **P (%)** | **P/T** | **P (%)** |
| **20** | 78/78 | 100 | 78/78 | 100 |
| **10** | 78/78 | 100 | 78/78 | 100 |
| **5** | 78/78 | 100 | 78/78 | 100 |
| **2** | 72/78 | 92,3 | 69/78 | 88,5 |
| **1** | 49/78 | 62,8 | 42/78 | 53,8 |
| **0.5** | 30/78 | 38,5 | 25/78 | 32,1 |
| **0.1** | 7/78 | 9,0 | 6/78 | 7,7 |

P: positive results numbers; T: total test numbers; P (%): positive rate.

**Table 15 Analysis of results of the collaborative trial for *POD* test**

| **Parameters** | | **Porcine** | **Chicken** |
| --- | --- | --- | --- |
| Number of laboratories | | 13 | 13 |
| Number of PCR replicates per dilution level | | 6 | 6 |
| *POD* curve | The mean probability of detection across laboratories (*LPOD*) | 0,78 | 0,74 |
|  | 95% confidence interval for *LPOD* | 0,67-0.87 | 0,63-0,84 |
|  | Slope b relative to the ideal *POD* cure (b=1) | 1,17 | 1,18 |
|  | Laboratory standard deviation σ_L_ | 0,30 | 0,30 |
| *LOD_95%_* [in copies] | Theoretical median laboratory | 3,1 | 3,3 |
